# N-glycosylation of infectious bronchitis virus M41 spike determines receptor specificity

**DOI:** 10.1099/jgv.0.001408

**Published:** 2020-03-26

**Authors:** K. M. Bouwman, N. Habraeken, A. Laconi, A. J. Berends, L. Groenewoud, M. Alders, V. Kemp, M. H. Verheije

**Affiliations:** ^1^​ Division of Pathology, Department Biomolecular Health Sciences, Faculty of Veterinary Medicine, Utrecht University, Utrecht, The Netherlands; ^†^​ Present address: Department of Comparative Biomedicine and Food Science, University of Padua, Legnaro (PD), Italy

**Keywords:** N-glycosylation, virus–host interactions, infectious bronchitis virus, coronavirus, spike protein

## Abstract

Infection of chicken coronavirus infectious bronchitis virus (IBV) is initiated by binding of the viral heavily N-glycosylated attachment protein spike to the alpha-2,3-linked sialic acid receptor Neu5Ac. Previously, we have shown that N-glycosylation of recombinantly expressed receptor binding domain (RBD) of the spike of IBV-M41 is of critical importance for binding to chicken trachea tissue. Here we investigated the role of N-glycosylation of the RBD on receptor specificity and virus replication in the context of the virus particle. Using our reverse genetics system we were able to generate recombinant IBVs for nine-out-of-ten individual N-glycosylation mutants. *In vitro* growth kinetics of these viruses were comparable to the virus containing the wild-type M41-S1. Furthermore, Neu5Ac binding by the recombinant viruses containing single N-glycosylation site knock-out mutations matched the Neu5Ac binding observed with the recombinant RBDs. Five N-glycosylation mutants lost the ability to bind Neu5Ac and gained binding to a different, yet unknown, sialylated glycan receptor on host cells. These results demonstrate that N-glycosylation of IBV is a determinant for receptor specificity.

## Introduction

Infectious bronchitis virus (IBV) is an enveloped, positive-strand RNA virus, belonging to the genus *Gammacoronavirus* in the order of *Nidovirales*. IBV is endemic in most countries around the world and causes huge economical losses in the poultry industry. Many different IBV strains circulate worldwide, which were recently classified into 32 phylogenetic lineages (G-I 1–27, G-II, GIV-GVI) [1]. Viruses of the historically first identified IBV Massachusetts group (Mass, including the prototype IBV-M41 and attenuated derivative IBV-H52) are now typed as G-I-1, which in Europe is currently the third most prevalent genotype [2]. IBV-Mass primarily infects the ciliated epithelial lining of the respiratory tract of chickens and infection leads to clinical symptoms including sneezing, coughing and snicking [3].

The virion of IBV is composed of four structural proteins, including the nucleocapsid (N) protein, encapsidating the genomic RNA, and the envelope proteins membrane (M), envelope (E) and spike (S). The S protein of IBV is a homo-trimer, which is translated as a single polypeptide and subsequently cleaved by host proteases to generate two subunits, S1 and S2. These subunits remain non-covalently bound in the virion, where S2, the stalk of the spike, is anchored in the membrane and essential for virion–host membrane fusion. The S1 subunit forms the head of the spike protein, which is essential for receptor binding. IBV strains use (alpha-2,3-linked) sialic acids [4, 5] for viral attachment to host cells. For IBV-M41 in particular Neu5Acα2-3Galβ1-3GlcNAc (Neu5Ac) has been identified as its glycan receptor [6].

Spikes of coronaviruses are heavily decorated with glycans on asparagine (N) residues (N-glycosylation, consensus sequence N-x-S/T), and conserved across different genotypes [7–10]. Glycosylation of the spike in human coronaviruses proved a determinant for protein folding [8, 9], receptor interactions [11] and can mask neutralizing epitopes present on the spike, thereby affecting the host immune system [12]. To support, a single amino acid substitution in a N-glycosylation site in the receptor binding domain (RBD) of SARS-CoV resulted in reduced binding to the host defense peptide mannose-binding lectin, thereby potentially affecting the immune reaction after SARS-CoV infection [12]. For IBV-M41, the cryo-EM structure revealed 20 asparagine residues are decorated with N-glycans present on the spike [13], of which nine are present on the M41-RBD. Furthermore, others have shown that specific N-glycosylation mutations in the spike of a cell-adapted, non-pathogenic IBV-Beaudette strain result in delayed replication in Vero cells [7]. Recently, we showed using recombinantly expressed IBV-M41 RBDs (composed of the N-terminal domain of the S1 protein [14]) that at least nine-out-of-ten N-glycosylation consensus sequences in the RBD are indeed decorated with a variety of glycans [15]. Single N-to-A mutations of each of these N-glycosylation sites demonstrated that glycosylation of M41-RBD at six different positions (N33, N59, N85, N126, N160 and N194) were essential for binding to its receptor Neu5Ac in glycan ELISA and for binding to chicken trachea tissues [6]. Here we set out to study whether the effects observed using recombinant proteins is reflected by recombinant virions when the same mutations are introduced in the S1 protein of IBV-M41.

To assess the role of N-glycosylation of the spike of pathogenic IBV-M41, we set out to rescue recombinant viruses using our previously published targeted recombination system [16], where the *S1* of vaccine strain H52 was replaced by that of IBV-M41 (r-M41-S1) or by individual N-glycosylation mutants thereof (r-M41-N-#-A). While nine-out-of-ten recombinant viruses could be rescued, showing similar replication kinetics in chicken embryo kidney (CEK) cells compared to r-M41-S1, the receptor specificity of five mutants was changed, based on glycan ELISA and haemagglutination assays using chicken erythrocytes. This reveals that N-glycosylation of S1 in the virion has implications on receptor binding and specificity.

## Methods

### Construction of plasmids

To obtain the fragment coding for the *M41-S1* gene, RNA was isolated from a virus stock of IBV-M41 (Animal Health Service, The Netherlands) using the QIAamp viral RNA Mini Kit (Qiagen, Germany). Reverse transcription was performed using the Transcriptor First Strand cDNA Synthesis Kit (Roche, Switzerland) with random hexamers according to the manufacturer’s protocol. PCR was performed with Phusion Hot Start II High-Fidelity DNA Polymerase (Thermo Fisher Scientific) using the primers listed in Table 1. To exchange the *S1* domain of IBV-H52 for that of M41 in the plasmid used for targeted recombination, the *M41-S1* PCR product and the previously generated plasmid containing the *H52-S* gene [16] were digested using restriction enzymes *Pac*I and *SnaB*I (both New England Biolabs, USA), ligated and subsequently transformed in HB101 *E. coli*. Sequences were confirmed by automated nucleotide sequencing (Macrogen, The Netherlands). A step-wise ligation approach was used to obtain the *H52 M41-S1* donor plasmid. To introduce mutations leading to individual N-to-A substitutions in N-glycosylation sites in the receptor binding domain (RBD) sequence, site-directed mutagenesis was performed using the primers listed in Table 1. The sequences of the plasmids containing the *S1* gene with the introduced mutations were confirmed by automated nucleotide sequencing (Macrogen, The Netherlands).

**Table 1.** Primers used to generate the fragment coding for *M41-S1* and to introduce the point mutations, which lead to individual N-to-A substitutions at the indicated positions

Final construct	PCR performed on:	Forward primer	Reverse primer
p*-H52-M41-S1*	cDNA IBV-M41	GTCTTTAAT**TTAATTAA**GTGTGG	CCATTAGTGATTTT**TACGTA**AAACTGGTTCTC
p*-M41-N33A*	p*-H52-M41-S1*	ATTTCTAGCGAATCTAATAATGCAGGCTC	GCTGCTTCTTCTATAGCTATGACGG
p*-M41-N59A*	p*-H52-M41-S1*	GCTGCTTCTTCTATAGCTATGACGG	AACAACACGACCACCATGAATAGTAC
p*-M41-N85A*	p*-H52-M41-S1*	CTTTGCAGATACTACAGTGTTTGTTAC	GCACAGTGTGCAGTACAAAACTGAC
p*-M41-N126A*	p*-H52-M41-S1*	GCTTTAACAGTTAGTGTAGCTAAG	ATAGAAAAGCTGGCCATTTTTCATAG
p*-M41-N145A*	p*-H52-M41-S1*	TTTAACATCCGCATATTTAAATGGTG	GCATTAACACACTGAAATGATTTAAAAG
p*-M41-N160A*	p*-H52-M41-S1*	CTGCGACCACAGATGTTACATCTG	CAGAGGTGTAAACAAGATCACCATTTAAATATAC
p*-M41-N194A*	p*-H52-M41-S1*	TGGTACTGCACAAGATGTTATTTTG	GCAACAAAATAAGCCAGGGCTTTAAC
p*-M41-N219A*	p*-H52-M41-S1*	GCTTTTTCAGATGGCTTTTATCC	GCCAGTATTATACTGGCATGCTAAC
p*-M41-N229A*	p*-H52-M41-S1*	CTAGTAGTTTAGTTAAGCAGAGTTTATTG	CAATAAAAGGATAAAAGCCATCTG
p*-M41-N246A*	p*-H52-M41-S1*	CTACTGCTTTTACGTTACACAATTTC	CAACACTATTTTCACGATAGACAATAAAC

Nucleotides in bold indicate the restriction site sequences used to insert *M41-S1* PCR product into plasmid *H52-S*. Underlined nucleotides indicate codon mutations that result in asparagine to alanine changes.

### Rescue of the recombinant viruses

Targeted recombination was performed as described before [16]. For each recombinant virus that was rescued, two independent clones were produced. Nomenclature used for the recombinant viruses is r-M41 throughout, with r-M41-S1 for the wild-type *M41-S1* sequence (identical to accession number ABI26423.1) and r-M41-N-#-A representing N-glycosylation mutants, all in the IBV-H52 genetic background (*S* gene sequence of H52 identical to accession number ARJ35791.1 except for three amino acids (A132E, S678T and I687T). The rescued r-M41 viruses were passaged twice in 8-day-embryonated chicken eggs (ECE) until embryo death was observed (maximum of 7 days post inoculation). Allantoic fluid was harvested and the sequence of the *S1* gene was assessed by automated sequencing (Macrogen, The Netherlands). Virus stocks were subsequently produced by inoculation of passage 2 (P2) virus (diluted 1 : 10000) in 8-day-ECE, and allantoic fluid was harvested 24 h later to use for subsequent experiments. Viral quantity was calculated based on RT-qPCR, as described previously [16]. Primers were used at a final concentration of 300 nM each with the iTaq universal SYBR Green one-step kit (Bio-Rad Laboratories, Hercules, CA, USA). A dilution series of IBV-M41 was used as a reference for quantification.

### Immunohistochemistry

Anti-IBV immunohistochemistry on chorioallantoic membranes (CAM), collected in P0, was performed as described previously [16] using a monoclonal antibody against IBV S2 (Prionics, Thermo Fisher, USA).

### Growth kinetics on CEK cells

CEK cells were isolated from 18-day-ECE [17]. Single-cell suspensions were made and seeded in 24-well plates at 1.5*10^5^ cells per well onto glass coverslips. CEK cells were infected in triplicates with 1.5*10^6^ virus particles based on the calculated RT-qPCR titre. Culture media samples were collected at 8, 24, 48 and 72 h post infection (p.i.), after which RNA was isolated and RT-qPCR was performed in duplicates as previously reported [16] using a dilution series of IBV-M41 as a reference for quantification. Statistical analysis was performed using one-way ANOVA with Dunnett's multiple comparisons test at each individual time point against r-M41-S1. * indicates significant difference of *P*<0.05 between r-M41-S1 and IBV-M41wt.

### Immunofluorescence staining

CEK cells grown on coverslips were analysed by immunofluorescent staining as described previously [16]. Antibodies used were primary MAb 48.4 anti-nucleocapsid (CVI, The Netherlands) and secondary antibody Alexa Fluor 488 conjugate goat anti-Mouse IgG (H+L) (Invitrogen by Thermo Fisher Scientific) both diluted in PBS/5 % (v/v) normal goat serum.

### ELISA

Synthetic Neu5Acα2-3Galβ1-3GlcNAc-polyacrylamide (GlycoNZ, Russia) (0.5 µg per well) was coated in a 96-well maxisorp plate (NUNC, Sigma-Aldrich) overnight at 4 ˚C. This was followed by a blocking step using 3 % (v/v) BSA (Sigma-Aldrich, Germany) in PBS/0.1 % (v/v) Tween. Virus dilutions were made in triplicate and added to the plates, followed by incubation for 2 h at room temperature. Where indicated, virus stocks (10^6^ genome equivalents) were pre-treated with 50 mU ml^−1^ of Arthrobacter ureafaciens neuraminidase (AUNA) (Sigma-Aldrich, Germany) for 1 h at 37 °C, before application to the coated plates and incubated for 2 h at room temperature. After washing with PBS, primary monoclonal mouse anti IBV-S1 (104.1, CVI, The Netherlands) was applied in phosphate buffered Normal Antibody Diluent (NAD) (VWR Immunologic, USA) for 1 h at room temperature. Dako Envision HRPO labelled polymer anti-mouse (Dako, by Agilent Technologies, USA) at a 1 : 1 ratio (v/v) in NAD was added to the wells, and after 30 min the wells were washed with PBS. TMB buffer (3,3′,5,5′-tetramethylbenzidine, Thermo Scientific, USA) substrate was used as peroxidase substrate to visualize binding, after which the reaction was terminated using 2 N H_2_SO_4_. FLUOstar Omega (BMG Labtech) was used to measure optical densities (OD450_nm_) and MARS Data Analysis Software for analysis of the data.

### Haemagglutination assay

Chicken erythrocytes (chRBC) were collected from the brachial vein and collected in 3.2 % (v/v) sodium citrate (NaC) (VACUETTE, Greiner Bio-One, USA). chRBC were washed four times (1500 r.p.m. for 10 min) in PBS without calcium chloride and magnesium chloride (PBS-/-) (Sigma-Aldrich, Germany) and diluted in PBS-/- to a concentration of 2 % (v/v). Virus stocks (10^5^ genome equivalents) and/or chRBC’s were pre-treated with 5 mU ml^−1^ of AUNA (Sigma-Aldrich, Germany) for 1 h at 37 °C before twofold serial dilutions of the virus were made in in PBS-/-. chRBC were applied to the (pre-treated) viruses in a 1 : 1 vol ratio. Haemagglutination titres were determined after 15 min incubation at room temperature.

## Results

### Recombinant viruses with N-to-A substitution in the RBD can all, except for N126A, be rescued

**Fig. 1.**
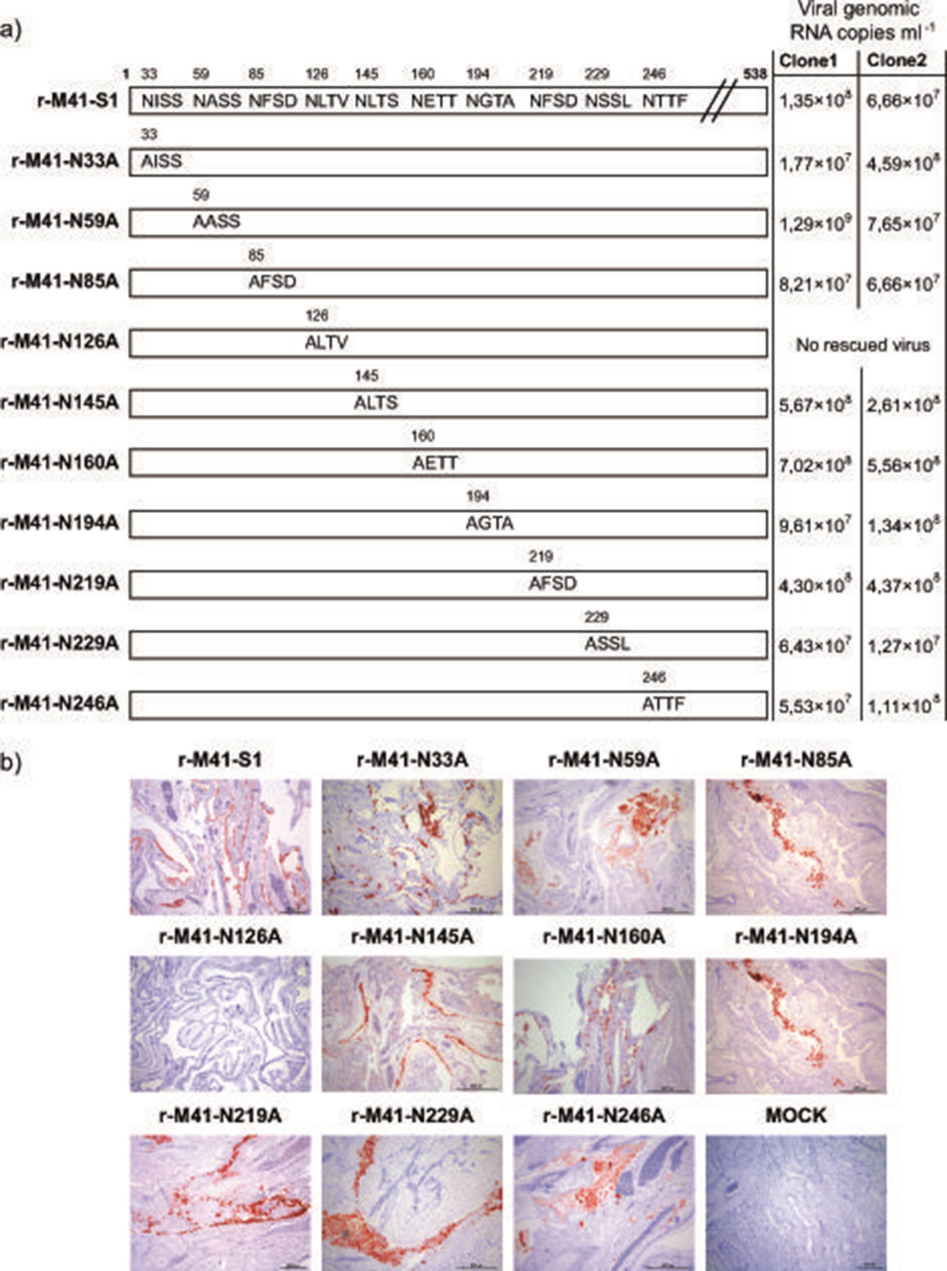
Generation of recombinant IBV with M41-S1 or N-glycosylation knock-out mutation in IBV-H52 background (r-M41-S1, r-M41-N-#-A). (a) Schematic overview of the M41-S1 protein including the N-glycosylation sites present in the RBD of the wild-type and the introduced N-to-A substitutions in the N-glycosylation knock-out mutants (numbering starts with amino acids ALY of the mature protein, signal peptide not shown). The numbers above the schematically represented mature wild-type M41 protein indicate all positions of the asparagine (N) substituted by alanine (A) residues. (b) Immunohistological staining of IBV-S2 in CAMs after inoculation with r-M41-S1 and r-M41-N-#-A mutants collected after targeted recombination (P0). Mock was inoculated with non-electroporated LR7 cells. Clone 1 is shown of each mutant, no differences between the two individual clones of each mutant were observed.

To study the role of N-glycosylation of the spike of IBV-M41 in the context of the virion, we generated ten N-glycosylation knock-out variants of the receptor binding domain (RBD). To this end, in the plasmids used to generate recombinant IBV-H52, the *S1*-coding sequence of IBV-H52 was replaced by that of wild-type IBV-*M41-S1*, or by *M41-S1* in which point mutations in individual N-glycosylation sites were introduced, resulting in asparagine (N) to alanine (A) substitution (schematic *S1* representation in Fig. 1a). Targeted RNA recombination was performed using the previously established system [16] and recombinant viruses were rescued on ECEs. All viruses, except r-M41-N126A, could be rescued, as confirmed by the detection of viral protein expression in the chorioallantoic membranes (CAM) of inoculated eggs (Fig. 1b, representative picture of clone 1). In four independent targeted recombination experiments, the rescue of r-M41-N126A was unsuccessful, as no viral protein expression in CAM tissue was observed (Fig. 1b), as well as no viral genomes being detected in the allantoic fluid by RT-qPCR of P0 (data not shown). For each rescued N-glycosylation knock-out mutant, two independent clones were isolated, passaged twice on ECE, after which the *S1* gene was sequenced to confirm the presence of the N-to-A substitution and to verify that no additional mutations were introduced. Virus stocks were grown in ECE after which by RT-q-PCR the viral quantity was determined ranging between 1.27×10^7^ and 1.29×10^9^ viral genomic RNA copies ml^−1^ (Fig. 1a), and used in subsequent experiments.

### N-glycosylation of the RBD does not affect viral infection and replication in CEK cells

**Fig. 2.**
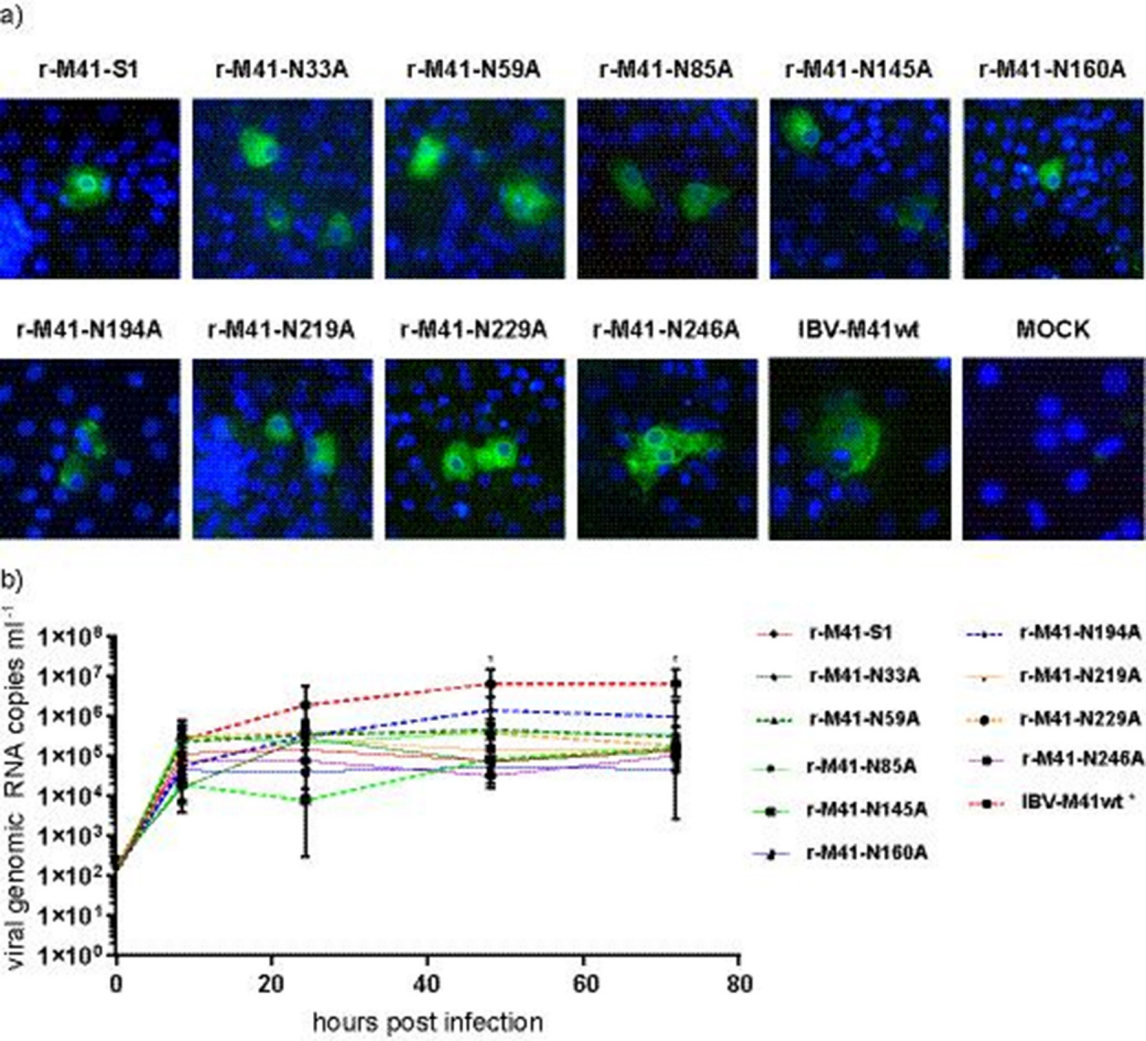
Infection and replication of recombinant IBVs on CEK cells. (a) Immunofluorescent staining of CEK cells inoculated with wild-type IBV-M41, r-M41-S1 or N-glycosylation mutants (N-#-A) using an antibody against the nucleocapsid protein. (b) Growth kinetics of wild-type IBV-M41 and recombinant IBVs assessed by RT-qPCR analysis. RNA was extracted from cell-culture supernatant collected at 8, 24, 48 and 72 h p.i. Data points represent means and standard deviations of the triplicates. Clone 1 is shown of each mutant. * indicates significant difference *P*<0.05 of IBV-M41 compared to r-M41-S1.

To compare the growth kinetics of the rescued recombinant viruses, CEK cells were inoculated with r-M41-S1 and each of the rescued N-glycosylation mutants. After 8 h, infection of CEK cells was confirmed by observing viral nucleocapsid protein expression for all viruses in immunofluorescent staining (Fig. 2a, representative picture of clone 1). The growth kinetic of all the viruses was assessed determining the relative viral load in the CEK culture supernatant at different time points post infection (Fig. 2b, clone 1 is shown). All recombinant viruses grew to titres not significantly different from r-M41-S1 (Fig. 2b), with the exception of clone 2 of r-M41-N145A (not shown), which had a titre around 1×10^2^ throughout the course of infection, whereas titres of r-M41-S1 were between 1×10^4^ and 1×10^5^ from 8 h p.i. onwards for both clones. IBV-M41wt was included as a positive control, which showed a significant higher viral load at 48 and 72 h p.i., potentially explained by the chimeric nature of the genetic background of the recombinant viruses and IBV-M41wt. These results show that all rescued recombinant viruses were able to enter CEK cells, and replication in CEK cells appears not to be affected significantly by N-glycosylation of the RBD of the IBV spike.

### Five IBV N-glycosylation mutants lost affinity for Neu5Ac

**Fig. 3.**
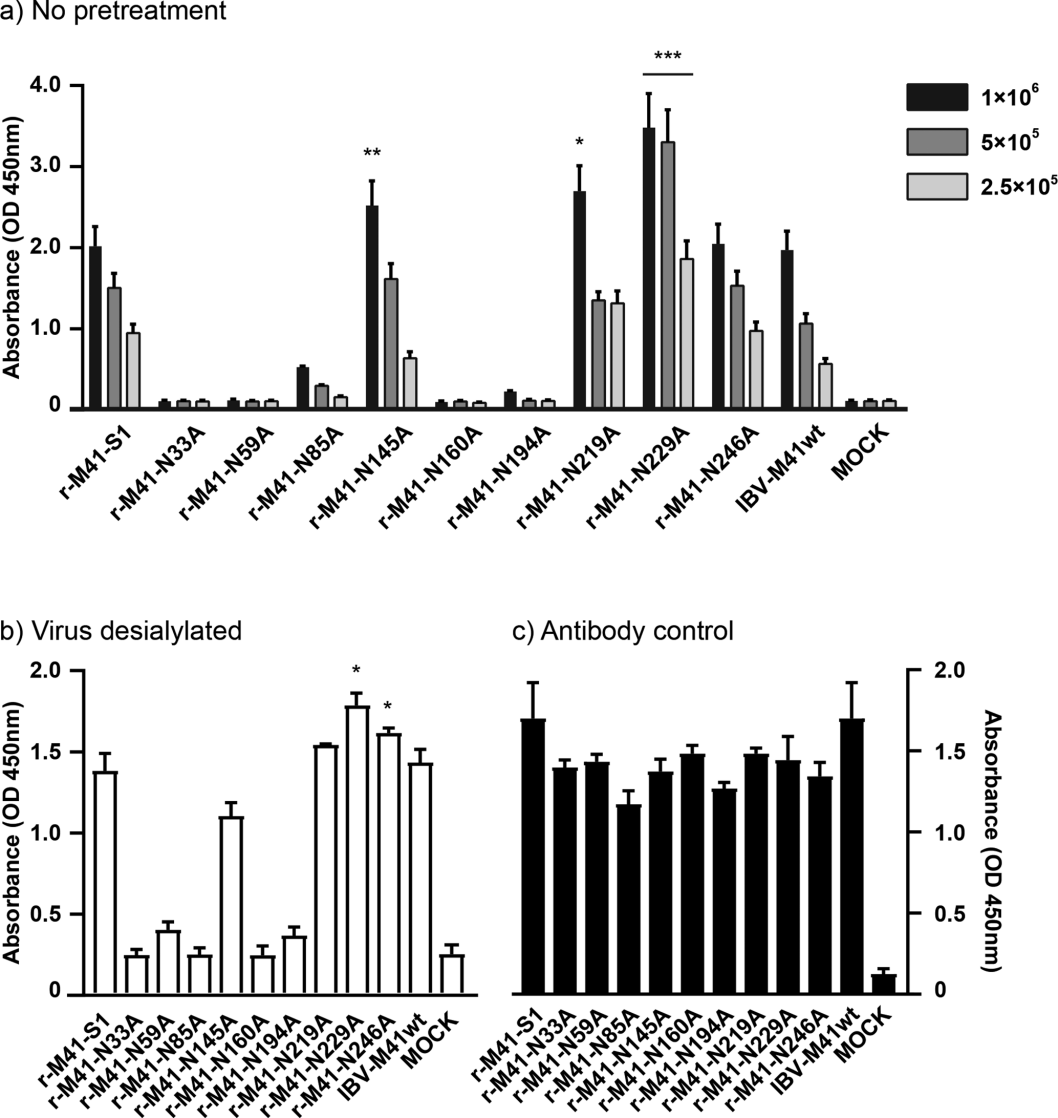
Binding of recombinant IBVs to Neu5Ac. (a, b) Affinity of wild-type IBV-M41, r-M41-S1 and N-glycosylation mutants thereof to synthetic Neu5Ac2-3Gal1-3GlcNAc-polyacrylamide (Neu5Ac) in solid phase ELISA, where in (b) all viruses were pre-treated with neuraminidase (1×10^6^ viruses applied). Bars represent means and standard deviations of the triplicates. Significant differences were calculated (at each virus amount) using one-way ANOVA where *P<0.05, **P<0.01 and ***P<0.005 indicate significant higher affinity compared to r-M41-S1. (c) ELISA plate coated with r-M41 or wild-type IBV-M41 to determine the antibody specificity for the different r-M41 mutants. Clone 1 is shown of each mutant, no significant differences between the two individual clones of each mutant.

To assess whether the rescue of nine-out-of-ten recombinant viruses was based on infection through binding of Neu5Ac, we investigated whether N-glycosylation mutants in the viral context retained affinity for this receptor. r-M41-N145A, N219A, N229A and N246A, were still able to bind Neu5Ac in a concentration-dependent manner, whereas for r-M41-N33A, -N59A, -N85A, -N160A and -N194A no binding to Neu5Ac was observed (Fig. 3a, clone 1 shown). We observed a significantly higher avidity of r-M41-N229A for Neu5Ac at all applied concentrations when compared to r-M41-S1, while r-M41-N145A and r-M41-N219A showed a higher avidity only at the highest concentration (Fig. 3a). Interactions to Neu5Ac were not dependent on sialylation of the viruses, since pre-treatment of the viruses with neuraminidase did not prevent binding to Neu5Ac (Fig. 3b, clone 1 shown). To confirm that the antibody used to detect the recombinant viruses is not dependent on epitopes affected by N-glycosylation site mutations, the ELISA plate was coated with the different r-M41 viruses and absorbance of the antibody was measured. This resulted in the recognition of all viruses, albeit slightly higher for both viruses containing wildtype S1 (r-M41-S1 and IBV-M41wt) (Fig. 3c, clone 1 shown).

### r-M41-N33A, -N59A, -N85A, -N160A and -N194A recognize a different sialylated receptor compared to r-M41-S1.

**Fig. 4.**
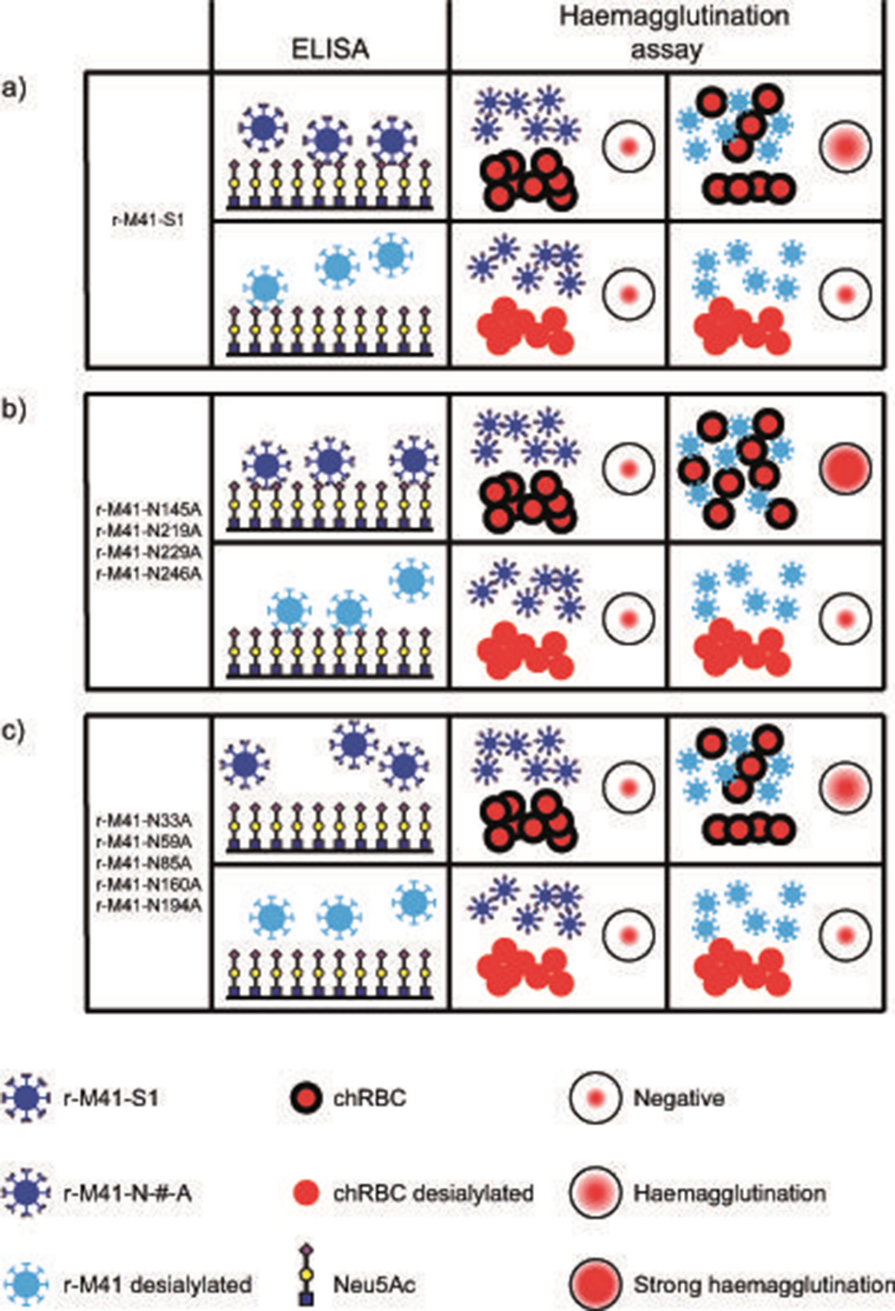
Graphic summary of the interactions between the recombinant IBVs and Neu5Ac in ELISA, and chRBC in haemagglutination assays, for (a) r-M41-S1, (b) r-M41-N145A, -N219A, -N229A and -N246A and (c) r-M41-N33A, -N59A, -N85A, -N160A and -N194A. Recombinant viruses are schematically represented in dark blue (containing diamonds indicating sialic acids on the virion) or light blue (sialic acids removed). chRBCs were untreated (schematically represented as red circles including thick black outline) or pre-treated to remove sialic acids (red circles without outline). Results are schematically represented side views of the assays, except for the haemagglutination read-outs (top views).

To demonstrate and confirm the N-glycosylation mutants unable to bind Neu5Ac are dependent on a different (glycan) receptor, haemagglutination assays were performed using chicken erythrocytes (chRBC). These host cells display a variety of glycans on their surface [18], and are used in diagnostic tests for serotyping IBV, where IBV needs to be pre-treated before haemagglutination is observed [19]. While r-M41-S1 had a haemagglutination titre of 32 (Table 2, first column and Fig. 4a), higher titres were observed for all r-M41 viruses that were still able to bind Neu5Ac in ELISA (Table 2, first column and Fig. 4b). All mutants that lost the ability to bind Neu5Ac in ELISA (r-M41- N33A, N59A, N85A, N160A and N194A) showed comparable haemagglutination titrer to that of r-M41-S1 (Table 2, first column and Fig. 4c), confirming a functional ligand binding site is present in these mutants. As expected, no haemagglutination using any of the r-M41 viruses was detected when no pre-treatment of the viruses was applied (Table 2, second and fourth column and Fig. 4). To further study the involvement of sialic acids as ligands involved in virus–host binding, chRBC were pre-treated with commercial neuraminidase prior to the haemagglutination assay. Removal of sialic acids with neuraminidase completely prevented haemagglutination, indicating that haemagglutination in all cases is dependent on the presence of sialic acids on the chRBC (Table 2, last two columns, and Fig. 4).

**Table 2.** Haemagglutination titres of r-M41-S1 and N-glycosylation mutants of chicken erythrocytes (chRBC) without and with pre-treatment of the virus and/or chRBC with neuraminidase

Pre-treatment virus	+	−	+	−
**Pre-treatment chRBC**	**−**	**−**	**+**	**+**
r-M41-S1	32	0	0	0
r-M41-N33A	32	0	0	0
r-M41-N59A	32	0	0	0
r-M41-N85A	32	0	0	0
r-M41-N145A	512	0	0	0
r-M41-N160A	64	0	0	0
r-M41-N194A	16	0	0	0
r-M41-N219A	256	0	0	0
r-M41-N229A	512	0	0	0
r-M41-N246A	1024	0	0	0

Taken together, we observed for r-M41-S1, as well as for r-M41-N145A, -N219A, -N229A and -N246A binding to Neu5Ac in ELISA (Fig. 3), independent of the presence of sialic acids on the virion (Fig. 4a, b) ELISA schematics, light and dark blue virions binding to Neu5Ac). Interestingly, no binding to Neu5Ac was observed when r-M41- N33A, N59A, N85A, N160A and N194A were applied, suggesting that receptor specificity of those viruses has changed compared to the wild-type (Fig. 4) ELISA schematic, light and dark blue virions binding to Neu5Ac). In haemagglutination assays, binding to chRBC could only be observed when the virus particles, but not the chRBC, were desialylated (Table 2 and Fig. 4 haemagglutination schematics). These results show that binding specificity can be determined using a synthetic ligand in ELISA. However, on living cells desialylation of the virions may be required to allow binding to a sialylated host receptor, of which the nature for r-M41- N33A, N59A, N85A, N160A and N194A is yet to be resolved.

## Discussion

Here we reveal that N-glycosylation of at least five sites in the spike protein of IBV-M41 affects the binding specificity for sialylated host glycans. The ability of recombinant viruses to recognize Neu5Ac is completely in line with binding of recombinant RBD proteins in ELISA [15], indicating that specific interactions and receptor specificity can be studied using recombinant proteins alone. In particular, r-M41 viruses containing substitutions at positions 33, 59, 85, 160 and 194 are dependent on a different sialylated glycan receptor than Neu5Ac for virus–host interactions. Importantly, r-M41-N229A and at higher concentrations r-M41-N145A and N219A, bound with higher affinity to Neu5Ac compared to r-M41-S1. These results may in part explain the higher haemagglutination titres observed using these mutants. Interestingly, r-M41-N246A, the mutant showing the highest haemagglutination titre (Table 2), bound with equal avidity to Neu5Ac in ELISA (Fig. 3a). This may point towards simultaneous binding to a different sialylated receptor present on chRBCs. Interestingly, the observed differences in receptor specificity or affinity do, however, not result in differences in the ability of these viruses to enter, replicate or produce infectious virions in primary chicken embryo kidney cells. The viruses do contain a functional receptor binding site as they all induce haemagglutination and we were able to rescue them.

Nine-out-of-ten single substitutions in N-glycosylation sites in the spike protein of IBV allowed rescue of recombinant viruses. We cannot draw any definitive conclusion for the reason why r-M41-N126A could not be grown in embryonated eggs, but some speculations can be addressed. Previously, we observed that recombinantly expressed RBD-N126A lost its binding to chicken trachea tissue; however, this cannot be the sole explanation, as the same was observed for five other mutants (N33A, N59A, N85A, N160A and N194A) [15], which apparently are able to bind a different sialic acid receptor now that they are introduced into the virion. In an earlier study [7], it was shown that mutation of glycosylation site N144 (in our study N126), and N163 (in our study N145), of IBV-Beaudette resulted in lower viral titres in Vero cells compared to wild-type, suggestive that the glycosylation site at position 126 might be directly involved in receptor binding. In contrast to IBV-M41, IBV-Beaudette can bind to heparan sulphate [20], and this might explain the ability to rescue IBV-Beaudette in cell culture, despite N-glycosylation mutations introduced in the spike at position 144.

Recently the cryo-EM structure of IBV-M41 was resolved, revealing the complete spike of IBV-M41 is decorated with N-glycans to (at least) 20 asparagine residues [13]. In our previous study using recombinant M41-RBD proteins, we successfully showed at least nine-out-of-ten consensus sequences present in the M41-RBD are decorated with N-glycans (all except N219) [15]. Eight-out-of-ten N-glycosylations we detected on the recombinant M41-RBD are in agreement with the findings in the resolved cryo-EM structure [13]. In our study using recombinant M41-RBD, we did not detect N-glycans on position N219, potentially due to inadequate amount of input material. The cryo-EM study did not identify N-glycans on N246, so the natural occurrence of these N-glycosylation sites remains to be verified. In the present study we chose to include both sites and these N-to-A mutations (N219A and N246A) did not result in loss of affinity for the known ligand (Fig. 3a), suggesting these sites are not directly involved in Neu5Ac binding. For other coronaviruses including Bovine CoV (BCoV) and Human CoV-OC43 (HCoV-OC43), both dependent on host glycan receptors containing terminal 9-O-acteylated sialic acids, the ligand binding sites are resolved and for both charged amino acids (BCoV: E182 and H185, HCoV-OC43: K81) and amino acids containing a ring structure (BCoV: Y162 and W184, HCoV-OC43: W90) are directly involved in ligand binding [21, 22]. While the ligand binding site of IBV-M41 is not yet resolved, *in silico* ligand binding predictions previously point towards involvement of amino acids S87, N144 and T162 of the M41-RBD [15]. These amino acids are neither charged or contain a ring structure. However in the cryo-EM structure of IBV-M41, charged amino acids (D88, E161 and D164), and F86 containing a ring structure, are found near the predicted ligand binding site. Importantly, the predicted ligand binding site of IBV-M41 is surrounded by N-glycosylation sites N59, N85 and N160, which are all critical for Neu5Ac binding in our present study. We can only speculate on the exact ligand interaction for IBV-M41 and how N-glycosylation of the virion affects ligand binding specificity. These recombinant viruses may potentially show an altered tissue tropism or phenotype *in vivo,* which remains to be revealed.

The infectivity and growth kinetics of the recombinant viruses were not affected by N-to-A mutations in the RBD. Whereas wild-type IBV-M41 showed significantly higher titres than r-M41-S1, possibly due to its slightly different genetic background, the mutants showed titres comparable to r-M41-S1 at all timepoints (Fig. 2b). These results may be explained by the expression of different glycans on the cells used in this study, as we propose that at least two glycan receptors (Neu5Ac and an unknown receptor) are involved in binding of the r-M41 viruses to host cells. Since we were able to rescue the r-M41 viruses, analyse the growth kinetics and perform haemagglutination assays, we propose that both receptors are expressed on CAM tissue, CEK cells and chRBC. Whether the unknown receptor is expressed on trachea tissue is still an open question; however, when testing recombinant RBD proteins no binding to chicken trachea tissue was observed using RBD-N33A, -N59A, -N85A, -N160A and- N194A [15], suggesting absence or low expression of the unknown receptor in this tissue.

Many viruses are decorated with N-glycans on the virion surface, including the flaviviruses Dengue and Zika virus, influenza A virus, Ebola virus, HIV, Nipah virus and human coronaviruses [23]. The glycans are added upon biosynthesis of the viral proteins, using the host-cell machinery. Consequently, these N-glycans on the viral surface can mimic self-antigens [24] or mask neutralizing epitopes present on the virus attachment proteins, potentially leading to evasion from the host immune response [24]. For HIV, broadly neutralizing antibodies have been found in infected patients that target epitopes created by glycans present on the attachment protein, strongly suggesting that the viral ‘glycan shield’ can be important for vaccine development [25]. We can only speculate these recombinant IBV viruses with altered N-glycosylation potentially express novel glyco-epitopes leading to broadly neutralizing antibodies protecting against IBV infection. As shown in the present study, the reverse genetics system developed in our laboratory represent the ideal tool to investigate the function of N-glycosylation on the IBV spike, however the biological consequences of IBV spike glycosylation remains to be studied.
